# Supplementary material for: Epidermal Resident Memory T Cell Fitness Requires Antigen Encounter in the Skin
Source: bioRxiv. 2025 Jul 9:2025.03.31.646438. Originally published 2025 Apr 5. Preprint. [Version 2] doi: 10.1101/2025.03.31.646438 (PMC11996394; doi:10.1101/2025.03.31.646438)

Supplemental Material for

Epidermal Resident Memory T Cell Fitness Requires Antigen Encounter in the Skin.

Eric S. Weiss[1,2], Toshiro Hirai[3,4], Haiyue Li[1,2], Andrew Liu[1,2], Shannon Baker[1,2], Ian Magill[5,6], Jacob Gillis[1,2], Youran R. Zhang[1,2], Torben Ramcke[1,2], Kazuo Kurihara[1,2] ,The ImmGen Consortium OpenSource T cell Project, David Masopust[7], Niroshana Anandasabapathy[8], Harinder Singh[2,9], David Zemmour[5,6], Laura MacKay[10], Daniel H. Kaplan[1,2]

Supplemental figure 1: (A) Quantification of percent $CD103^+$ T cells from VV and DNFB treated flanks, gated on live $CD45.2^+CD8^+CD90.2^+$. Each bar represents the mean +/- SEM for n= 6 animals. (B) Gating strategy for skin single-cell RNA-seq. (C) Flow plots of CD69 and CD103 expression of VV and DNFB treated flanks for single-cell RNA-seq gated as in (B). (D) Heatmap showing the top 10 DEG per cluster when compared with each other, calculated by FindAllMarkers of Seurat V2, percent expressed > 0.02, Log2foldchange > 2.

Weiss et al. Supplemental Figure 1

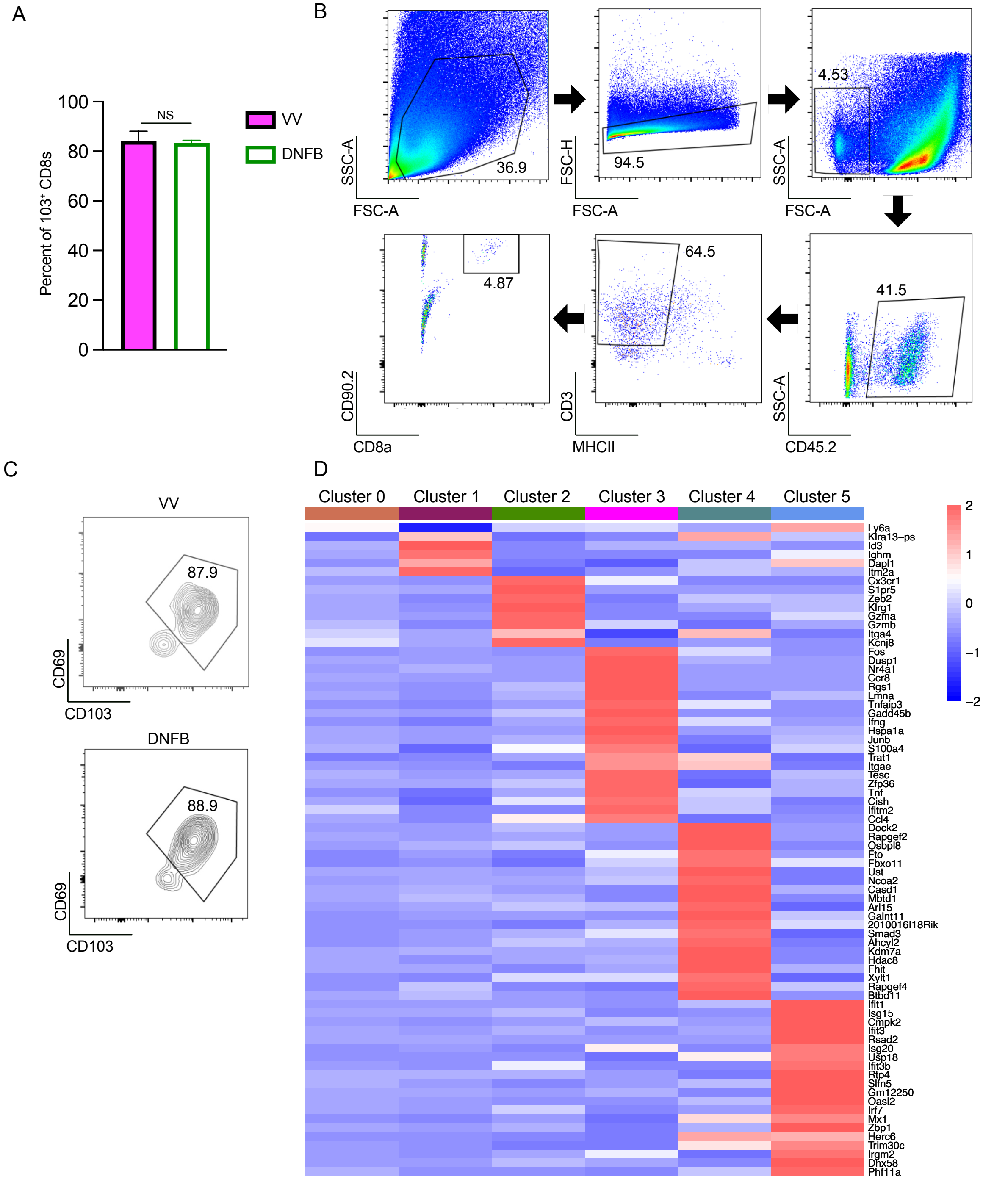

Supplemental figure 2: (A) Representative flow plots of blood from Thy1.1 OT-I adoptive transferred mice after 6 days of vehicle or FTY720 treatment, gated on liveCD45$^+$. (B) Quantification of total Thy1.1 OT-I in blood after FTY720 treatment, gated as in (A). Each bar represents the mean +/I SEM for n= 6 animals. (C) Representative flow plots of blood of CD45$^+$CD3$^+$CD8$^+$ cells after isotype or Thy1.1 depleting antibody treatment. (D) Quantification of Thy1.1 OT-I in blood before and after isotype or anti-Thy1.1 depleting antibody treatment, gated as in (C). (E) Representative epidermal whole mount of Thy1.1$^+$ cells in the epidermis at steady state of VV-OVA or DNFB treated flanks after 6 days of isotype or anti-Thy1.1 depleting antibody treatment. Each symbol represents data from an individual animal. Data is representative of 3 separate experiments. *$p<0.05$ by unpaired Student's t-tests. Scale bar represents 50um.

Weiss et al., Supplemental Figure 2

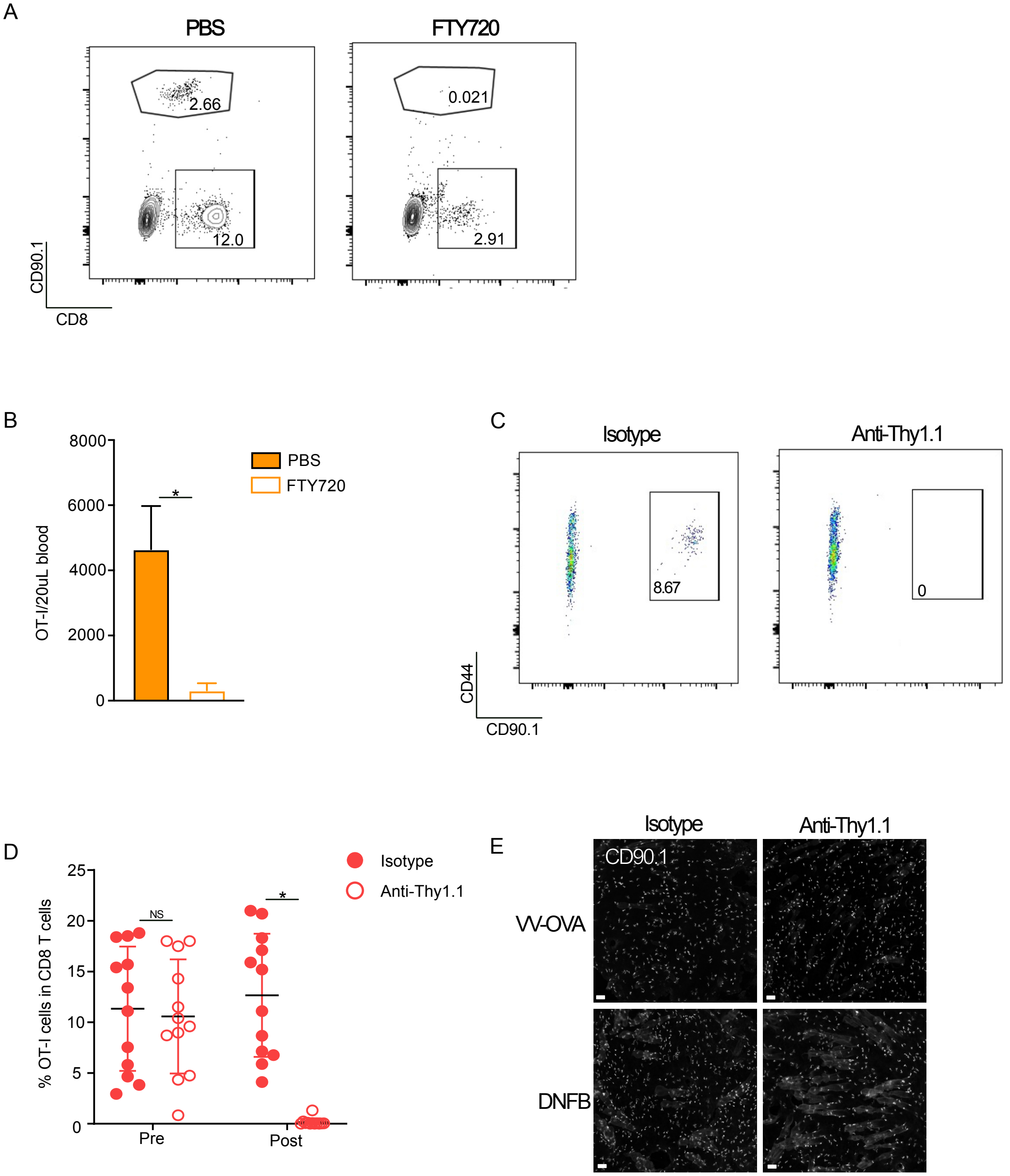

Supplement: Supplement 1 [file media-1.pdf]
